# Perioperative platelet rich plasma (PRP) in total hip arthroplasty through the Hardinge approach: protocol to study the effectiveness for gluteus medius healing

**DOI:** 10.1186/s40634-018-0127-7

**Published:** 2018-06-19

**Authors:** Anni Aavikko, J. Puhakka, J. Haapala, J. Kukkonen, K. Mäkelä, J. Kosola

**Affiliations:** 1 Department of Orthopaedics and Traumatology, Helsinki University Hospital, Helsinki, Finland; 2 Department of Surgery, Satakunta Central Hospital, Pori, Finland; 3 Department of Orthopaedics and Traumatology, Turku University Hospital, Turku, Finland

## Abstract

**Background:**

Platelet-rich plasma (PRP) has been used to support tendon regeneration mainly in sports medicine. PRP is a concentrate of platelet-rich plasma proteins derived from whole blood by centrifugation to remove erythrocytes and leukocytes. PRP has high amounts of platelets which may promote healing tendons affected by degenerative conditions. These platelets contain growth factors and are known to facilitate the regeneration of injured tendon structures. Total hip arthroplasty (THA) through the Hardinge approach may leave the patient with impaired gait and poor regeneration of the gluteus medius tendon if the tendon is not reattached properly after closure of the surgical wound.

**Methods:**

The study will be a multicenter, double-blinded and randomized study enrolling 90 patients based on power calculations. The efficacy of perioperative PRP treatment will be assessed by subjective and objective outcome variables. The participants will be randomized (sealed envelope) into either a placebo (saline) or a PRP group (1:1). For subjective outcomes, the Oxford Hip Score (OHS) will be collected before surgery and 3 and 12 months after surgery. The objective measures are findings at magnetic resonance imaging and plain radiographs and recorded values of measured strength.

**Discussion:**

We present the perioperative use and the ways to measure the clinical efficacy of PRP. As PRP may have benefits regarding degenerative tendon regeneration, studies on the use of PRP in hip arthroplasty are warranted to facilitate postoperative recovery.

**Trial registration:**

This study has been approved by the ethics committee of the Hospital District of Southwest Finland and approved by the local institutional research board. The study has been registered in ClinicalTrials.gov (NCT02607462).

## Background

Total hip arthroplasty (THA) is a common orthopedic procedure (Kurtz et al. 2007). In Finland, over 8000 hip arthroplasty procedures have been made annually since 2012 due to osteoarthritis (OA) (Finnish Arthroplasty Register 2015). Recently, an American arthroplasty register study found that the total distribution of arthroplasty procedures in the United States was 860,080 of which 32,2% (277,200) were total hip arthroplasties (AJJR annual Report 2017). Kurz et al. estimated that by 2030, the demand for primary THA in the US will grow by 174% to 572,000 (Kurtz et al. 2007) and this increasing trend seems to be a worldwide phenomenon (Kurtz et al. 2010).

Two commonly used approaches in THA are the Moore approach (posterior approach) and the modified Hardinge approach (direct lateral approach) (Pellicci et al. 1998; Hardinge 1982; Finnish Arthroplasty society 2015; Madsen et al. 2004). Other possible approaches are the Smith-Petersen direct anterior approach and the Watson-Jones anterolateral approach (Smith-Petersen 1949; Watson-Jones 1934). Chechik et al. surveyed 292 orthopedic surgeons from 57 countries and found that the posterior approach was used by 45% and the direct lateral approach by 42%. The anterior approach was used by 10% of the surgeons and other approaches by 3% (Chechik et al. 2013).

Six months after THA up to 85% and 2 years after THA up to 10% of patients may experience abduction weakness of the hip muscles (Madsen et al. 2004; Mulliken et al. 1998). This complication seems to be associated with the surgical approach used. When the Hardinge approach is used, the gluteus medius and minimus muscles are split to allow anterior dislocation of the hip; at the end of the operation, these muscles are attached to their insertion (Mulliken et al. 1998). Residual abductor and limb weakness may result from avulsion of the repair of the anterior portion of the abductors or from direct injury to the superior gluteal nerve. The occurrence of these complications (positive Trendelenburg sign and abnormal gait) is reportedly no less than 11% (Havelin et al. 2016; Ramesh et al. 1996). When the posterior approach is used and the gluteus medius remains intact, these complications can be avoided (Petis et al. 2015). Different approaches lead to different complications (Petis S et al. 2015). A well-known risk of the posterior approach is femoral dislocation, the rate of which is 1–5% (Petis et al. 2015; Jolles et al. 2006). Careful reconstruction of the joint capsule and short external rotators may decrease the risk of postoperative dislocation (Kwon et al. 2006).

Platelet rich plasma (PRP) is used in various areas of surgery to enhance bone and soft-tissue repair by close proximity of supraphysiological concentrations of autologous platelets at the site of tissue damage. The α granules of platelets are rich in growth factors that are essential for tissue repair: transforming growth factor-β, vascular endothelial growth factor and platelet-derived growth factor (Alsousou et al. 2009). The interaction between these growth factors and surface receptors on the target cells activates intracellular signaling pathways that induce the production of proteins needed for the regenerative processes, such as cellular proliferation, matrix formation, osteoid production and collagen synthesis (Schliephake 2002).

PRP can be prepared in a laboratory, the operating room or clinic room immediately before surgery. There are three methods for preparation: gravitational platelet sequestration (GPS), standard cell separation and autologous selective filtration (thrombapheresis) (Alsousou et al. 2009). Because PRP is prepared from autologous blood it is inherently safe. Any concerns associated with allografts or xenografts regarding transmission of diseases, such as HIV, hepatitis or Creutzfeld-Jakob disease, or of immunogenic reactions are eliminated (Man et al. 2001).

The increased awareness of the effectiveness of PRP in musculoskeletal healing has expanded its indications in orthopedic and sports medicine (Fitzpatrick et al. 2017). A randomized controlled study reported that intra-articular PRP injections offer significant clinical improvement for patients with hip osteoarthritis but is void of substantial side effects (Man et al. 2001). Topical PRP in total knee arthroplasty (TKA) reduces postoperative blood loss (Mochizuki et al. 2016; Aggarwal et al. 2014). PRP reduces pain after TKA (Aggarwal et al. 2014). On the other hand, a recent systematic review stated that the number of high quality RCT’s investigating the use of PRP in knee osteoarthritis is limited (Muchedzi et al. 2017). In a retrospective analysis there was no clinical benefit from the use of PRP in THA (Safdar et al. 2015). To our knowledge, no randomized controlled trials (RCT) have published on the effectiveness of PRP in musculoskeletal tissue healing at THA procedure.

In this paper, we describe the protocol of our ongoing randomized controlled trial assessing the effects of PRP in minimizing postoperative problems for patients who have undergone THA with the Hardinge approach.

## Methods and analysis

### Objectives and study hypothesis

The objective of this trial is to compare the healing of the gluteus medius insertion among patients who have undergone THA. We will operate patients through the Hardinge approach and inject PRP or placebo in the region of the gluteus medius tendon insertion. The primary objective is to investigate the healing of the gluteus medius insertion objectively with MRI (anatomy) and hip strength measurements (dynamometer). The secondary objective is to record and analyze the changes in the Oxford Hip Score (OHS) by comparing the OHS before and after surgery and to record and analyze clinical signs, especially limping, pain and movement restrictions. The tertiary objective is to analyze safety and the cost-effectiveness of PRP in THA. The hypothesis is that PRP facilitates postoperative healing of the gluteus medius among elderly patients after THA and that the healing can be verified objectively seen by MRI and dynamometry.

### Study design

**Fig. 1:**
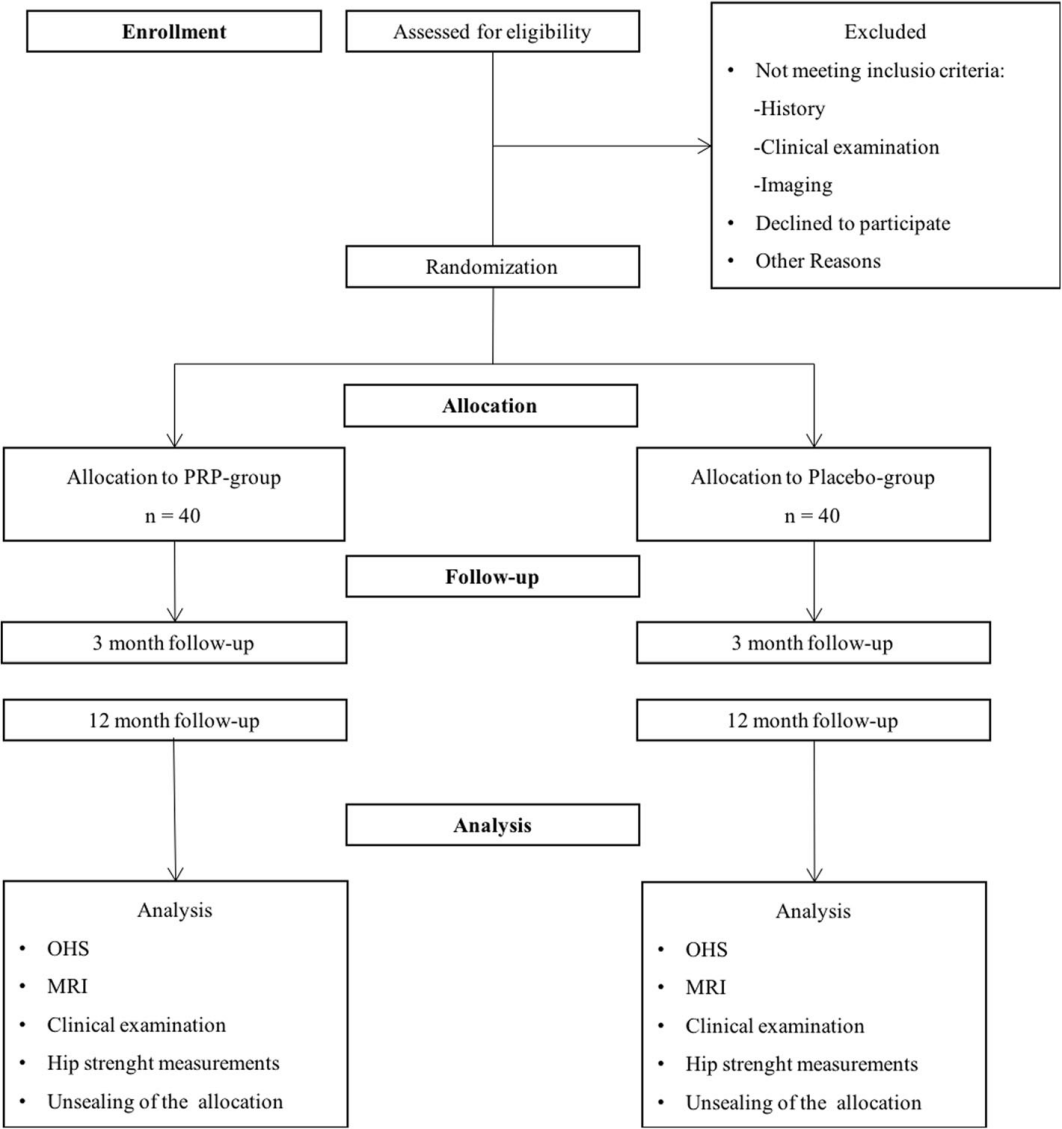
Flowchart of the trial: enrollment, treatment allocation and follow-up scheme

The study is a multicenter RCT with parallel study groups (1:1). The study design is double blinded where neither the orthopedic surgeon nor the patient know whether the patient receives PRP treatment or placebo. The flowchart of the study cohort is shown in Fig. 1 including enrollment, allocation, follow-up and analysis. After having provided written informed consent the patients are enrolled. They participate voluntary in the study, they may withdraw from the trial at any time and are not compelled to explain the reason for withdrawal.

Enrollment began with a pilot phase conducted in a secondary hospital in Finland, where the study protocol was followed. A total of 18 patients were recruited. The THA procedures was done by four orthopedic surgeons experienced in the Hardinge approach and in injecting PRP during closure. Postoperative open care visits to the same orthopedic surgeons who performed the THA were carried out and hip strength assessments were done by a trained physiotherapist.

### Patients

The patients will be recruited to the study from the THA surgery queue. Patients who have been referred in writing to the hospital (Päijät-Hämeen keskussairaala, Lahti, Finland) for THA will receive a written invitation to participate in the study. This invitation will be sent to the patient together with the invitation to attend for an open care visit at the hospital for preoperative assessment for the THA. During the this interview the patient discusses the treatment plan with the operating orthopedic surgeon, after which one of the study physicians (AA or JK) will provide detailed information about the study. Then a from for providing written informed consent is handed to the patient. After the interview patient will have 2 weeks time to get familiar with the study and consider participation in the study. After this, the patient will have the option to sign and submit the consent form or to decline to do so when arriving to the hospital for the THA.

The inclusion and exclusion criteria are shown in Table 1. Patinets aged 60–75 years are eligible. This age span has been decided upon based on the profile of the patients undergoing THA in Finland and who are eligible for cementless THA (Mäkelä et al. 2008). The other inclusion criteria (degree of osteoarthritis and clinical symptoms) will be determined by the orthopedic surgeon who decides if the THA procedure is to the probable benefit of the patient (Table 1). The exclusion criteria are based on the conceivable complications related to PRP or rehabilitation.

**Table 1:** Inclusion and exclusion criteria

Inclusion criteria (all of the following):	
1. Age 60–75 years	
2. Plain x-rays showing osteoarthritis (Kellgren and Lawrence gradus 3–4) which correlates with clinical symptoms	
3. Clinically symptomatic osteoarthritis requiring treatment with total hip arthroplasty	
Exclusion criteria (any one of the following):	
1. Previous surgery of the affected joint	
2. Post-traumatic arthritis	
3. Rheumatoid arthritis and other autoimmune illnesses	
4. Malignancy	
5. Patients on oral glucocorticosteroids	
6. Insulin-dependent diabetes	
7. Smoking	
8. Alcohol or drug abuse	
9. Mental instability	

### Preparation of PRP, placebo

For the preparation of PRP, 50 ml of fresh venous blood with 5 ml sodium citrate (anticoagulant) will be collected into a single syringe from an antebrachial vein. The collected venous blood will be separated into 4 × 10 ml PRP-syringes (GLO PRP kit™, Glotech Co, Korea) for centrifugation. The PRP will be done according the manufacturer’s instructions with 2 separate centrifugations: the first centrifugation will be for 5 min at 1200 rpm and the second for 2 min and 1200 rpm. After the first centrifugation, red blood cells will be collected and discarded. The second centrifugation will be used to concentrate the platelets and separate the buffy-coat from the PRP. From each PRP-syringe, 2.5 ml of concentrated PRP will be collected from the bottom of the syringe and 10 ml of PRP ready for injection will be produced. Of the 10 ml, a 1 ml sample will be taken for laboratory analysis. The placebo consists of saline, and has been found to be appropriate (Schöffl et al. 2017).

### Baseline

The primary OA diagnosis will be made by the orthopedic surgeon who assesses whether the patient benefits from THA. In support of this decision, the surgeon will have access to plain radiographs of the affected hip joint and information on the range of movements of the hip joint. Inclusion mandates that the patient has OA Kellgren-Lawrence grade 3–4. The strength of both hips at baseline will be assessed by a physiotherapist on the day of surgery and the baseline OHS will be calculated and recorded.

### Interventions

**Fig. 2:**
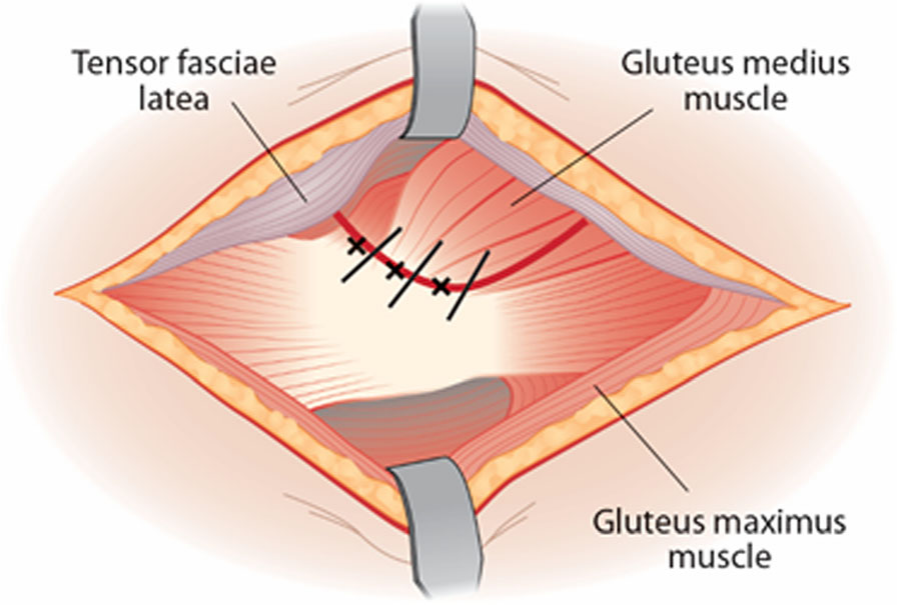
Injection site for PRP or placebo (marked with *). Black lines indicate the suturation via bone tunnels

The THA procedure will be done per routine so that the gluteus medius muscle is partly stripped from its trochanteric insertion during the approach to the hip joint. This stripped part of the gluteus medius muscle will be sutured back to the insertion site with non-absorbable sutures via bone tunnels at the closure phase of the procedure. The blinded placebo or PRP will be injected into the tendon incision line of the gluteus medius (Fig. 2) and targeted into the bone tendon interval. No leakage of PRP is expected after injection, since PRP will be injected at multiple sites after closure with bone tunnels and non-absorpable sutures (Ethibond nro. 1, Ethicon/ Johnson-Johnson).

The postoperative rehabilitation will follow national guidelines: the patient is mobilized with full weight bearing and free range of movements. The rehabilitation protocol is of beneficit to the patient (Tsukagoshi et al. 2014; Andersson et al. 2001). Cruches will be used primary for safe mobilization and a physiotherapist will give detailed instructions for postoperative rehabilitation to each patient.

### Randomisation and concealment

The final decision of inclusion or exclusion of the patient into the trial will be done during the THA procedure where the surgeon will evaluate the condition of the gluteus medius. If the gluteus medius seems abnormal (i.e., torn or atrophied), the patient will be withdrawn from the study. If gluteus medius is intact, the surgeon will notify the staff nurse who will open a sealed envelope. The sealed envelopes will be kept in a secure and agreed location known only to the trial physicians and nurses; the surgeon will not know which group the participant is included. If the patient is allocated to receive placebo, 50 ml of blood will be drawn but the blood will not be prepared for PRP. The venous sample will be taken and PRP prepared by a study physician (AA or JK) who will not participate the THA surgery. Instead, after about 30 min the surgeon will be given a blinded (taped) 10 ml syringe with saline by the instrument nurse.

If the patient is randomized to receive PRP, 50 ml of blood is drawn, as for the placebo procedure. This blood will be prepared in an operation room behind the surgeons back. Then the surgeon will be given a blinded (taped) 10 ml syringe with PRP.

### Outcome measures

To measure objectively the healing of the gluteus medius and to check the positions of the components of the joint prosthesis, MRI and plain radiographs will be taken 3 and 12 months postoperatively. At the same control visits, a trained physiotherapist will measure the hip strengths and the OHS will be recorded. Clinical examinations will be conducted (movements of both hip joints, wound healing) and any limping or a positive Trendelenburg sign will be recorded.

### Oxford hip score

Patients will fill OHS forms preoperatively and postoperatively at 3 and 12 months after surgery.

### Magnetic resonance imaging - MRI

MRI sequences will be used according to the MARS protocol (Pfirrmann et al. 2005), where T1 sequences will be used to identify gluteus medius pathology, and coronal and sagittal images are taken. The imaging time per sequence is about 3–6 min and the entire MRI study will take approximately 30 min in total.

### Clinical examination – gait and Trendelenburg test

The Trendelenburg test and the hip lag sign will be examined 3 and 12 months after the THA by the surgeon. The hip lag sign is tested by putting the patient or his side with the operated hip upwards and by asking the patient to abduct the examined extremity. This testing position mimics the validated hip strength measurements used in the present study protocol and is shown below.

### Hip strength measurements

**Fig. 3:**
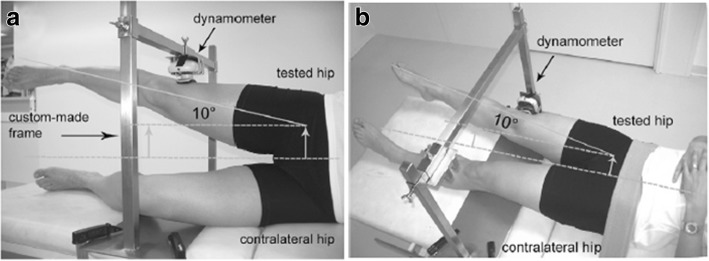
Dynamometric hip strength measurement (Widler et al. 2009)

To test the reattachment and function of the detached gluteus medius required by the Hardinge approach, hip abduction strength will be measured with a dynamometer. The abduction forces of both lower extremities will be tested with the patient lying supine and on his side (Fig. 3).

### Reoperations, loss to follow-up and missing data

All reoperations, loss to follow-up and missing data will be recorded. Patients who experience perioperative or postoperative complications related to healing of the gluteus medius (i.e.*,* trochanteric fracture or infection) will be censored from the final analysis. In order to minimize loss to follow-up and missing data, the dates for the postoperative visists will be set and given in writing to the patients already at the ward; this includes the visit for strength assessment and clinical examinations. MRI and radiography will be done during the same week as the 3- and 12-month clinical control visits.

### Data management and statistical analysis (sample size)

Due to multiple outcome settings, the OHS was used as the primary outcome measure and power calculations were carried out accordingly. Thus, a sample size of 40 patients per intervention group has been calculated as being sufficient when a 5 point mean difference (SD 7.8 point) is used to identify a significant difference at a significance level of 0.05 and 80% power (two-sample t-test).

### Ethics

The etics committee of the Hospital District of Southwest Finland approved the study (ETMK 77/2015, 21.3.2017).

### *Single* versus *multiple surgeons/centers*

Although single-center studies in RCTs has been met with criticism (Moseley et al. 2002), one of the main purposes of the pilot study protocol was to generate a logistically feasible setting which could be easily transferred to any other primary, secondary or tertiary hospital in Finland. As we want to know only the healing of the gluteus medius, objective methods of follow-up (MRI and strength assessment) will be used.

## Pilot study results

Our pilot study results have been presented previously at the German Congress of Orthopaedics and Traumatology 2017 (DKOU 2017) (Puhakka et al. 2017). A total of 12 patients participated in the pilot study in the Satakunta Central Hospital, Finland. The OHS of these patients increased in 3 months by 23.5 points in the PRP group compared to an increase of 20.2 points in the group who got placebo. The PRP group had a higher average increase in the maximum strength abduction compared to those who got placebo (39.1 vs. 6.2%). These preliminary results were in line with the power analysis, where significant differences (*p* < 0.05) are expected with 40 patients per study groups. The basic charasteristics of the pilot study group are given in Table 2.

**Table 2:** Basic characteristics of pilot study groups

Characteristic	PRP *n* = 6	Placebo *n* = 6
Age, mean (range)	66.5 (63–72)	70.3 (68–73)
Gender (M / F)	3 / 3	5 / 1
Height (cm)	175 (165–183)	170 (156–179)
Weight (kg)	81.4 (72–90)	78.0 (63–87)
BMI	26.4 (25.5–26.9)	27.1 (21.3–30.1)
Illnesses		
Type 2 DM, oral medication	1	1
High blood pressure medication	3	–
Coronary artery disease	–	1
Hypercholesterolemia	–	1
Platelet-rich plasma characteristics		
Number of cells per patient (×10^6^, mean, 9 ml)		
Red blood cells	0.11	–
Leukocytes	1.10	–
Platelets	60.0	
Concentrations (× 10^9^, mean)		–
Red blood cells	0.12	–
Leukocytes	1.21	–
Platelets	66.7	

## Discussion

The present study protocol uses has several point-of-care methods in an attempt to reveal if a single perioperative PRP injection into the reinsertion site of the gluteus medius during THA benefits the patient. Firstly, we will analyze MR images to examine the anatomy and healing of the reattached gluteus medius. MRI is of high clinical value in symptomatic THA patients who have a compromised gluteus medius muscle (Pfirrmann et al. 2005). MRI can also reveal significant abductor atrophy after THA (Engelken et al. 2014; Roth et al. 2014). Secondly, OHS data will be collected for a detailed understanding of the overall patient outcome after THA. Previously OHS has been compared to various hip scores and it has been validated as a registry tool (Nilsdotter et al. 2001). It has been shown that a change in the total score of 4.85 is clinically meaningful (Beard et al. 2015). Thirdly, clinical examination with special attention to any gait abnormalities will be included. Historically, the Trendelenburg sign has been used to diagnose gluteus medius injuries, but the reliability of this sign has been questioned (Youdas et al. 2010). Poor abduction strength seems to be associated with abnormal gait (limping). During gait, abduction of the lower extremity is produced by the gluteus medius muscle (Anderson et al. 2003). Kaltenborn et al. have created a clinical sign – the hip lag sign – which correlates with the degree of gluteus medius impairment in MRI (Kaltenborn et al. 2014). The reported specificity and sensitivity of the hip lag sign is excellent, 96.5% and 89.7%, respectively. In the present study the hip lag test will be carried out augmented by dynamometric measurements to assess the strength of the hip postoperatively. This method has been validated for hip abduction power measurements (Widler et al. 2009).

The need for THA will increase massively in the future and research to identify more rapid and cost-effective postoperative rehabilitation is needed. The present study plan describes a RCT aimed at determining if PRP benefits a given subgroup of THA patients.

### Limitations

The main limitation of the study will be the use on non-standardized PRP preparations. The amount of platelets in a batch of PRP cannot be determined during the procedure and controlling the concentration of platelets and leukocytes in a given batch of PRP is difficult (Shahid & Kundra, 2017). Indeed, there have been no studies which have solved the issue of how to homogenize fresh PRP. Because the quantity of leukocytes and platelets seems to be highly relevant, we will take a 1 ml control sample from the final injectable PRP and analyze it in the hospital laboratory. The control sample will be taken in an EDTA-containing test tube to prohibit coagulation and delivered to the laboratory within 30 min after sampling.

The study design does not allow distinguishing between muscle weakness due to the THA and damage to the superior gluteal nerve. Presistant symptoms and clinical signs of a positive Trendelenburg test due to the nerve injury is rare (Petis et al. 2015). To detect a positive Trendelenburg test reliably, we could have added postoperative gait assessment to the protocol, but since gait analysis is not routinely used in the clinic, we have not included this in the study protocol. No previous studies have used gait analysis usefully in settings similar to this stuidy.

## Conclusion

This paper summarizes how randomization will be carried out and describes the follow-up of patients with respect to postoperative healing of the gluteus medius muscle with objective and subjective methods. This placebo-controlled RCT is designed to answer the question of whether intervention with perioperative PRP applied in the area of the gluteus medius tendon insertion affects the outcome of patients who have undergone THA via Hardinge approach.
